# Evaluating Whole Grain Intervention Study Designs and Reporting Practices Using Evidence Mapping Methodology

**DOI:** 10.3390/nu10081052

**Published:** 2018-08-09

**Authors:** Caleigh M. Sawicki, Kara A. Livingston, Alastair B. Ross, Paul F. Jacques, Katie Koecher, Nicola M. McKeown

**Affiliations:** 1 Nutritional Epidemiology, Jean Mayer USDA Human Nutrition Research Center on Aging at Tufts University, Boston, MA 02111, USA; caleigh.sawicki@tufts.edu (C.M.S.); kara.livingston@tufts.edu (K.A.L.); paul.jacques@tufts.edu (P.F.J.); 2 Gerald J. and Dorothy R. Friedman School of Nutrition Science and Policy, Tufts University, Boston, MA 02111, USA; 3 Department of Biology and Biological Engineering, Chalmers University of Technology, 41296 Gothenburg, Sweden; alastair.ross@chalmers.se; 4 Bell Institute of Health, Nutrition and Food Safety, General Mills, Inc., Minneapolis, MN 55427, USA; katie.koecher@genmills.com

**Keywords:** whole grain, evidence map, intervention, reporting practices

## Abstract

Consumption of whole grains have been associated with reduced risk of chronic diseases in many observational studies; yet, results of intervention studies are mixed. We aimed to use evidence mapping to capture the methodological and reporting variability in whole grain intervention studies that may contribute to this inconsistency. We conducted a reproducible search in OVID Medline for whole grain human intervention studies (published 1946 to February 2018). After screening based on a priori criteria, we identified 202 publications describing a total of 213 unique trials. Over half (55%) were acute trials, lasting ≤1 day, 30% were moderate duration studies (up to 6 weeks) and 15% were of longer duration (more than 6 weeks). The majority of acute trials (75%) examined measures of glycaemia and/or insulinemia, while most of the longer trials included measures of cardiometabolic health (71%), appetite/satiety (57%) and weight/adiposity (56%). Among the moderate and long duration trials, there was a wide range of how whole grains were described but only 10 publications referenced an established definition. Only 55% of trials reported the actual amount of whole grains (in grams or servings), while 36% reported the amount of food/product and 9% did not report a dose at all. Of the interventions that provided a mixture of whole grains, less than half (46%) reported the distribution of the different grain types. Reporting of subject compliance also varied and only 22% used independent biomarkers of whole grain intake. This evidence map highlights the need to standardize both study protocols and reporting practices to support effective synthesis of study results and provide a stronger foundation to better inform nutrition scientists and public health policy.

## 1. Introduction

Whole grain foods have long been promoted as part of a healthy diet and since 2000, specific whole grain recommendations have been included in the Dietary Guidelines for Americans (DGA) [1]. The most recent 2015 DGA recommends consuming 6 ounce-equivalents of grains per day (based on a 2000 kcal diet) and that at least half of this amount should be whole grain [2], while some Nordic countries recommend 75 g whole grain per 10 MJ of energy [3]. While the majority of prospective studies has found that consumption of whole grains is associated with health benefits, including reduced risk of cardiovascular disease (CVD), obesity, diabetes and all-cause mortality [4,5,6,7], evidence of health benefits from intervention studies on obesity and cardiometabolic risk factors is less consistent. Variation in study design and diet intervention, including the type of grain(s) provided, dose, duration and background diet of the intervention makes it difficult to compare and synthesize the conclusions drawn from these studies. Furthermore, universal consensus in defining whole grain and especially what constitutes a whole grain food is lacking [8]. Definitions vary by different organizations, particularly for whole grain food products [9,10]. In 1999, AACCI (formerly the American Association of Cereal Chemists (AACC) International) released a definition stating, “Whole grains shall consist of the intact, ground, cracked, or flaked caryopsis, whose principal anatomical components—the starchy endosperm, germ and bran—are present in the same relative proportions as they exist in the intact caryopsis” [11]. This definition captures whole grain ingredients, such as flours and intact grains but does not define whole grain food products. The AACCI published that a whole grain food product should have ≥8 g of whole grains for every 30 g of product (i.e., the typical serving of a cereal product) [12], while the USDA requires food products have ≥8 g (dry weight) whole grains per reference amount customarily consumed and ≥51% of the grain ingredients must be whole grain [13]. In 2010, the Healthgrain Forum, a non-profit organization of academics and industry interested in cereals and health, issued a similar whole grain definition as the AACCI; however, this definition specifies “small losses of components, that is, less than 2% of the grain or 10% of the bran that occurs through processing methods are allowed” to account for what happens at most flour mills. More recently, the Healthgrain Forum has defined whole-grain foods as a food that “contains >30% whole grain ingredients in the overall product and contains more whole grain than refined grain ingredients, both on a dry weight basis,” and recommends that only foods that meet healthy eating criteria be labelled as “whole grain” [10]. Both the AACCI and Healthgrain definitions include the cereal grasses and the pseudocereals (amaranth, buckwheat, quinoa and wild rice). The 2015 DGA describes whole grains in terms of ounce-equivalents, with 1 ounce-equivalent of 100 percent whole grain food having 16 g of whole grains (based on the minimum whole grain content of a 1 ounce slice of bread that would meet the U.S. Food and Drug Administration (FDA) health claim criteria of 51% or more whole grain) [2]. Other definitions and recommendations use grams of whole grain. The lack of consistency and limitations in defining whole grain foods lends itself to confusion and creates difficulty in standardizing diet interventions and comparing results across intervention studies.

Recognizing the need to improve study design reporting in research in the whole-grain field, Ross and colleagues [14] highlighted several recommendations for reporting whole grain intake in both observational and intervention studies. These recommendations included the following: (i) report the grams of whole grain consumed/provided rather than the amount of whole grain food/product and account for water content; (ii) report which definition of whole grain is being used; (iii) report the proportion of different grains consumed/provided and not just the total amount of whole grains; (iv) describe the structure and processing of the grains; (v) report added bran or germ separately (a methodological issue encountered in observational studies); (vi) use a refined grain control instead of “usual diet”; (vii) use biomarkers of whole grain intake whenever possible.

We evaluated the differences in study design and reporting practices of studies, using the above criteria as guidelines, by creating an evidence map. Evidence mapping is a method by which scientific evidence on an expansive or complex topic is identified, organized and summarized [15]. Evidence mapping involves the collection of population, intervention, comparator and outcome (PICO) information in order to characterize the existing research landscape in a broad area. It is similar to the first steps in a systematic review; however, in contrast to systematic reviews, evidence mapping does not include data on study results or provide in-depth risk of bias evaluations. Instead, the focus is more on capturing the history and trajectory of the topic area, as well as an assessment of the variation in methodology, such as in the present study. One appreciable difference between evidence mapping and systematic reviews is that evidence mapping provides a larger, more comprehensive scope of the research area than is usually addressed in a systematic review and the goal is to produce an updatable descriptive map that can be used for different purposes. Evidence mapping has been used to promote evidence-based decision making [16,17,18] and this method has previously been employed to summarize research on nutrition topics such as sugar-sweetened beverages [19], dietary fiber [20], low-calorie sweeteners [15] and trans fatty acids [21]. 

The purpose of conducting an evidence map is to summarize research in the field, potentially providing researchers with insight about research gaps or trends that may facilitate new work in the field. The growing body of research on whole grains, the variation in outcomes in intervention studies, as well as the many health outcomes being measured with respect to whole grain intake, makes this an ideal area for using evidence mapping methodology. Therefore, the aim of this project was to (i) summarize the current body of literature on whole grain intervention studies and health and (ii) apply evidence mapping methodology to evaluate variation in study design and variation in reporting practices among longer-duration whole grain studies.

## 2. Materials and Methods

Evidence mapping consists of three main steps: (i) clearly defining a topic area of interest; (2) systematically searching and screening relevant studies based on pre-defined criteria; and (3) extracting and reporting on study characteristics or other questions of interest, creating a “map” of available evidence [15,16,17].

### 2.1. Search Strategy

We aimed to collect all published intervention studies examining the effect of whole grain consumption on health. We conducted a systematic, reproducible search in OVID Medline for whole grain intervention studies using a broad list of whole grain key terms. The search extended from inception of the Medline database (1946) to February 2018. The search was restricted to human intervention studies published in English. Observational studies, case reports, bibliographies, letters, editorials and reviews were specifically excluded. For the list of search terms used see Table S1. We used the multi-purpose search feature in OVID to search all fields including title, abstract, subject heading words and keyword heading words. 

### 2.2. Selection Criteria

Results of our search were screened in two phases based on a priori selection criteria. For phase 1, we used the free, open source platform, ABSTRAKR, developed by the Evidence-Based Practice Center at Brown University [22], to conduct an initial screening of abstracts identified in Medline. This phase used a low-threshold of inclusion in an attempt to include all potentially relevant publications. To be included at this phase, studies had to be (i) intervention studies; (ii) conducted in human subjects; and (iii) published in English. Exclusion criteria at this phase were: (i) animal studies; (ii) in-vitro studies; (iii) reviews, bibliographies, case reports, letters, or any other non-intervention study; (iv) no inclusion of whole grains in the intervention. Studies for which abstracts were not found or where inclusion/exclusion was unclear were moved forward for secondary screening.

For secondary screening, full-text manuscripts were obtained. During this phase, additional exclusion criteria were applied to better restrict the results to whole grain interventions most relevant to the general population. These exclusion criteria included the following: (i) the intervention did not allow the effect of whole grain to be isolated (including whole grain products with added bran); (ii) the intervention was only an isolated component of whole grain (e.g., bran, germ, fiber); (iii) subjects were following a gluten-free diet; (iv) subjects were <3 years old (purpose was complementary feeding practices with enriched whole grains on nutritional status or to assess digestibility of grain product in infants); (v) the study was specific to oral rehydration or refeeding therapies; (vi) subjects were pregnant or breastfeeding women; (vii) in vitro studies; (viii) animal studies. Reasons for exclusion during this phase were documented.

### 2.3. Data Abstraction and Analysis

An Excel spreadsheet was used to collate data from eligible publications. Information collected included study design, subject characteristics, details of whole grain interventions and controls (including dose, duration, processing and form) and reported outcomes (but not specific results). For studies that included more than one whole grain intervention group, a new record was created for each intervention. Some publications included multiple, distinct studies. These were entered as separate studies. To synthesize these data, studies were mapped descriptively by study design, intervention (whole grain type), and/or outcomes. These data can be used to create clusters of evidence in specific areas of focus.

Whole grain intervention treatments were extracted as described in the publications. We then grouped interventions by grain type (e.g., wheat, barley, rye). If the intervention included more than one type of grain given simultaneously (such as multigrain products or mixed whole grain diet interventions with commercially available products), or if the publication did not describe the specific type(s) of grains (e.g., fiber-rich whole-grains), these interventions were grouped under the general category “mixed whole grain (WG).” Similarly, control treatments were extracted as described in publications and were then grouped as refined grain, usual diet, matched (same intervention as treatment but without any added whole grain), other (including glucose, bran and unspecified controls), or no control arm.

We categorized studies by study duration as acute (<1 day), moderate duration (1 day to 6 weeks), or long duration (>6 weeks). We then categorized studies by design (randomized controlled parallel, randomized controlled crossover, or other designs), sample size, subject characteristics and region. We grouped outcome measures into the following broad categories: glycaemia/insulinemia (e.g., fasting and postprandial glucose and insulin, HbA1c, glucose turnover), appetite/satiety (e.g., hunger, fullness, energy intake, ghrelin), cardiometabolic health (e.g., blood pressure, lipids, inflammatory markers, oxidative markers), weight/adiposity (e.g., body weight, body mass index, waist-to-hip ratio, adipose tissue), gastrointestinal (GI) function/microbiota (e.g., transit time, laxation, fecal pH, bacterial composition, short-chain fatty acids, breath hydrogen), exercise/physical activity (e.g., resting metabolic rate, energy expenditure, physical activity, exercise intensity) and micronutrient status (e.g., blood and/or urine concentrations of sodium, potassium, magnesium, iron, zinc).

In addition to highlighting variability in study design, we also evaluated the variability in reporting practices in the moderate and long duration trials. For this purpose, we examined what types of whole grain definitions were used or referenced, how whole grain type and dose was reported and whether whole grain biomarkers were used for compliance. 

## 3. Results

### 3.1. Literature Search and Screening

**Figure 1:**
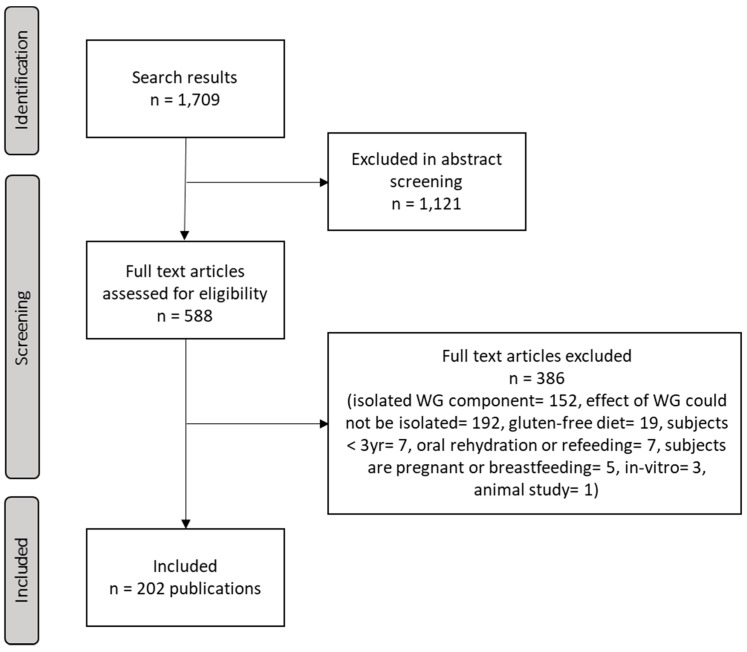
Flow diagram of literature search and screening results.

The results of the systematic Medline search for published intervention studies examining the effect of consumption of whole grains (1946 to February 2018) are presented in Figure 1. The initial search identified 1709 publications of which 1121 were excluded during abstract-level screening and an additional 386 were excluded during full-text screening. A total of 202 eligible publications were identified; however, because some of these publications described multiple unique trials, data was extracted from 213 trials. 

### 3.2. Study Design Characteristics

**Figure 2:**
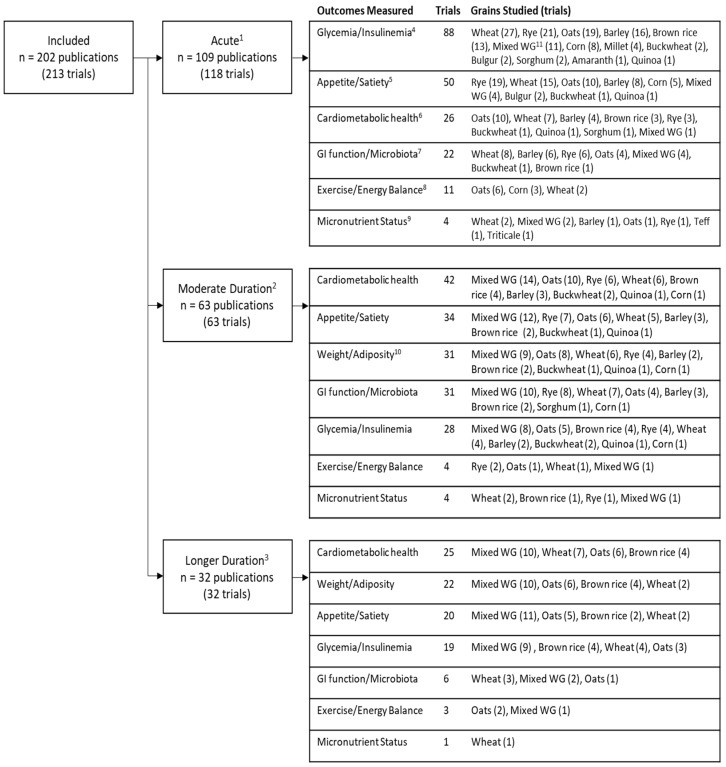
Outcome categories and grain types studied in whole grain intervention trials, organized by trial duration. ^1^ Acute: <1 day; ^2^ Moderate duration: >1 day to 6 weeks; ^3^ Longer duration: >6 weeks; ^4^ Glycaemia/Insulinemia e.g., fasting and postprandial glucose and insulin, HbA1c, glucose turnover; ^5^ Appetite/satiety e.g., hunger, fullness, energy intake, ghrelin; ^6^ Cardiometabolic health e.g., blood pressure, lipids, inflammatory markers, oxidative markers; ^7^ GI function/microbiota e.g., transit time, laxation, fecal pH, bacterial composition, short-chain fatty acids, breath hydrogen; ^8^ Exercise Performance/Energy balance e.g., resting metabolic rate, energy expenditure, physical activity, exercise intensity, stool energy density; ^9^ Micronutrient Status e.g., concentrations of sodium, potassium, magnesium, iron, zinc, minerals; ^10^ Weight/Adiposity e.g., body weight, BMI, body fat/adipose tissue, waist circumference, lean body mass; ^11^ Mixed whole grain (WG) group includes combinations of 2 or more whole grains, whole grain diets and unspecified “whole grain” foods (ex: whole grain bread).

Trials that lasted ≤1 day were labelled “acute” trials (*n* = 118 trials among 109 publications), while studies of moderate (>1 day to 6 weeks, *n* = 63 trials) and long (>6 weeks, *n* = 32 trials) durations are referred to as “longer duration” trials (*n* = 95 trials among 95 publications). Two publications detailed both acute and longer duration trials [23,24]. The number of trials by duration, with each major outcome category and the type of grain interventions, are summarized in Figure 2. The majority of the acute trials (75%, *n* = 88) focused on glycaemia and/or insulinemia outcomes, while the major outcomes in the longer (moderate and long) duration trials were cardiometabolic health measures (71%, *n* = 67), measures of appetite/satiety (57%, *n* = 54) and measures of weight/adiposity (56%, *n* = 53).

Table 1 summarizes the study design characteristics of all trials categorized by study duration (acute, moderate, long). Among the acute trials, the most common design was a randomized, controlled, crossover trial (*n* = 100, 85%). Only one trial used a randomized, controlled, parallel trial design and a number of studies did not specify randomization (*n* = 16), or used a single arm study design (*n* = 1). Most acute trials had a sample size of less than 25 (*n* = 97, 82%) and 33% had 10 or fewer subjects (*n* = 39). The majority of studies recruited healthy (*n* = 79, 67%) adults (≥18 years) (*n* = 115, 97%). Only 38% were metabolically-at-risk, that is, had type 2 diabetes (15%), were overweight or obese (16%), or had metabolic syndrome or at least one criteria of metabolic syndrome (hyperglycemia, hypertension, or hyperlipidemia) (7%). 

Moderate duration trials were also mostly RCTs with a cross-over design (*n* = 37, 59%), while long duration trials mostly implemented RCT parallel designs (*n* = 26, 81%). Of the other designs used, six trials were single arm (moderate duration) and one trial did not specify randomization (long duration). Moderate duration studies ranged from 2 days (*n* = 1 trial) to 6 weeks (*n* = 23 trials) (interquartile range (IQR) = 3 weeks). Long duration studies ranged from 8 weeks to 2 years (IQR = 3 weeks) but only 7 trials were > 12 weeks. Sample size ranged from 5 to 266 participants (mean ± SD = 45.7 ± 54.4) for the moderate duration trials and from 18 to 266 participants (mean ± SD = 92.3 ± 77.9) for the long duration trials. Similar to the acute studies, most of the longer duration trials were in adults (*n* = 91, 96%) but of the moderate duration 2 trials were conducted in adolescents (aged 12–17 years) and one in children aged 3–11 years. Compared to the acute studies, subjects recruited in the longer duration trials were less likely to be healthy; 40% were overweight or obese (*n* = 38) and 46% had other risk factors of the metabolic syndrome (*n* = 44).

**Figure 3:**
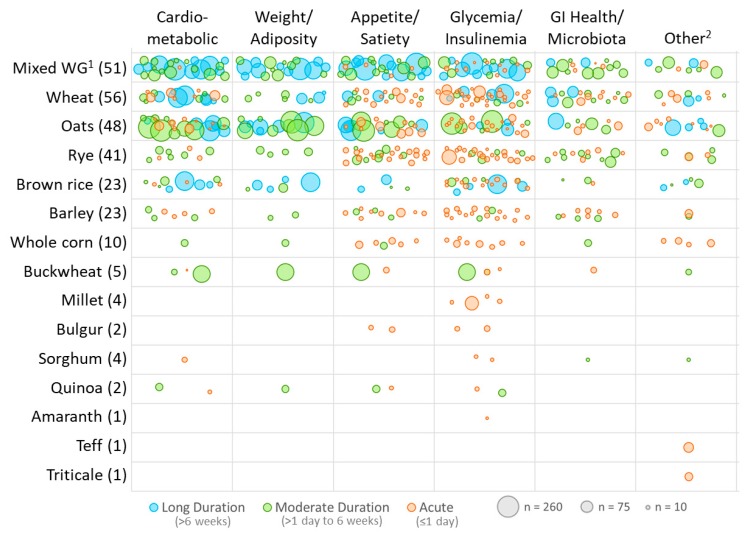
^1^ Each bubble represents a single publication within each grain by outcome category and the size corresponds to the study sample size; position of bubbles is random within each cell; trial bubbles are duplicated between outcome or grain categories when >1 outcome of grain type was studied; ^1^ Mixed Whole Grain (WG) group includes combinations of 2 or more WGs, WG diets and unspecified WG foods (ex: WG bread); ^2^ Other outcomes include exercise performance, micronutrient status, clinical blood and urine measures and other metabolites.

Figure 3 is a weighted scatter plot that displays the trials by the different grain types used in the interventions and the outcomes examined. Each bubble represents a single publication within each grain-by-outcome category, the size corresponds to the study sample size and the color corresponds to the duration of the trial (acute, moderate, long). Most trials administered a wheat-based intervention (21%), closely followed by mixed whole grain interventions (19%), oats (18%), rye (15%), barley (8%) and brown rice (8%). With respect to whole-grain diet interventions (grouped in mixed WG) (*n* = 39), the vast majority of the inventions provided study participants with the choice of commercially available whole-grain products (data not shown). The study design characteristics were similar across the top grain types, but, notably, none of the rye trials were of >6 weeks in duration or had larger sample sizes. In fact, only four grain types (mixed whole grains, wheat, oat and brown rice) were used in the long (>6 weeks) trials. The moderate duration trials additionally included rye, barley, buckwheat, sorghum and quinoa and just a few acute studies examined other types of grain (whole corn, millet, bulger, amaranth, teff and triticale). 

### 3.3. Reporting Practices

In Table 2, reporting practices based on recommendations from Ross et al., [14] for the moderate and long-term interventions were examined. The whole grain interventions were described in varying degrees of detail but most publications (*n* = 61, 73%) did not specify a definition for whole grain product or whole grain food. When a definition was provided, only 12% (*n* = 10) referenced an established definition, which included the American Association of Cereal Chemists (*n* = 5) [11,12], Proceedings of the Nutrition Society (*n* = 1) [25], Dietary Guidelines for Americans (*n* = 2) [26,27] and HealthGrain Forum (*n* = 4) [28]. The rest provided various general descriptions of whole grain or whole grain food (*n* = 12). Specific definitions/descriptions are listed in Table S2. When reporting the whole grain dose administered, the actual amount of whole grain provided was not always clear: 55% (*n* = 52) of longer duration trials reported grams or servings of whole grain but 36% (*n* = 34) reported the grams or servings of whole grain food/product (thus providing no actual quantifiable amount of whole grain) and 9% (*n* = 9) failed to report a dose at all. Further, among the longer interventions that provided a variety of whole grains (either as a part of a diet or a multi-grain food/product) (*n* = 39), less than half (46%, *n* = 18) reported the distribution of the different grains types. Additionally, while 73% used diet records or questionnaires to measure compliance, only 22% (*n* = 26) of longer duration trials used biomarkers of whole grain intake. A few (*n* = 14, 15%) used food weighing, wrappers, or direct observation and 20% (*n* = 19) did not report on any compliance measures.

## 4. Discussion

Until the last 10 years, there was little research on whole grains, with fewer than 6 whole grain intervention studies published per year prior to 2008 (Figure S1). With the rapid increase in studies, it is important to reflect on the current state and trajectory of the existing evidence. Using evidence mapping methodology, we are able to evaluate variation in study design and reporting practices among whole grain intervention studies. This evidence map consisted of 202 publications that described 213 unique whole grain intervention trials in humans. Acute trials (*n* = 118) consisted of a single meal or individual whole grain food (e.g., oats) intervention, with follow-up lasting up to 24 hours. These acute studies focused primarily on the postprandial effects of whole grain consumption, especially measures of glucose response and appetite/satiety. Moderate and longer duration trials (*n* = 95), in which whole grain interventions were given from 2 days to 2 years, captured dietary interventions that included a range of whole grain foods and whole-grain mixed diets (mainly based on wheat). The most frequent outcomes examined in the moderate and long duration studies included markers of cardiometabolic health (e.g., changes in lipids, markers of inflammation and oxidative stress, blood pressure) and changes in weight or adiposity, outcomes that have gained more focus particularly in the past 10 years (Figure S2). Gastrointestinal function and microbiota related outcomes have also gained more attention recently. With regard to grain type, whole grain wheat and oats have remained among the most frequently studied grain types among both acute and non-acute studies over time, but other grain types, including rye, brown rice, whole corn and buckwheat, have really only grown in interest over the last 10 years (Figure S3). Additionally, there has been a notable increase in mixed whole grain diet interventions since the early 2000s.

In terms of study design and subject characteristics, nearly all the acute trials used a cross-over design in healthy subjects, while a larger proportion of the non-acute trials utilized parallel arms and were more likely to include metabolically at-risk subjects. Most subjects in these trials were overweight or obese, had metabolic syndrome, or had other metabolic risk factors (hypertension, hyperlipidemia, hyperglycemia). However, there are far fewer trials of moderate and long duration, and, therefore, the literature may be unbalanced toward short studies among healthy subjects.

Another area of inconsistency in whole-grain research is the units used to report the whole grain quantity, or dose. Ross and colleagues [14] recommend that researchers report the gram amount of whole grains provided, rather than the amount of the whole grain food or product. In our evidence map, we found that while the majority (53%) of longer duration trials provided grams of whole grain (such as grams of barley kernels, grams of wheat flour, etc.), a large proportion (41%) instead reported the gram weight of the whole grain food (such as rye bread or muffin) or whole grain product (such as ready to eat breakfast cereal, packaged snack bars, etc.). Given the variability and limitations in defining a whole grain food (both among research publications and food industry) [10], the actual grams of whole grain ingredients delivered in the intervention could vary considerably because of the variation in the amount of whole grains in whole grain products, particularly when many whole grain products contain a mixture of whole and refined grain. This variability creates a challenge for researchers when attempting to estimate absolute whole grain intake in both observational and intervention studies [9,14] and confounds efforts to relate the amount of whole grains eaten to possible effects on disease risk. Similarly, the use of ounce equivalence in some studies, in-line with USDA dietary recommendations, also prevents accurate estimation of whole grain intake for the same reason that one ounce equivalent of whole grain food may be anywhere from 16 to 28 g.

Over 70% of moderate and long duration publications did not specify a definition of whole grain or whole grain food. It is important to note that many of these studies may have provided a single intact whole grain intervention, such as oats, and therefore may not have found it necessary to specify a definition of whole grain. However, when a whole grain is incorporated into foods (and diets) it becomes more important to specify these definitions. Although it seems that more recent studies are more likely to include a definition, the lack of a standard definition of whole-grain foods remains a major challenge for researchers, especially for comparing between different studies.

Interventions using a mixture of different types of whole grains, either in food products or overall diets, have become more prevalent over the past 5–10 years, particularly among non-acute trials (*n* = 39 trials). For example, some studies instructed subjects to consume a certain amount of whole grains per day, with a choice from a variety of products [29,30,31,32]. In such cases, Ross et al., have discussed the need to describe the proportion of each specific grain consumed/provided [14]. In our evidence map, we found that 54% of these mixed-grain intervention studies did not provide details on individual grain types. Different grains have different compositions, including the types and concentrations of fibers and phytochemicals [33,34,35] and, therefore, may have different physiologic effects. For example, there is some evidence that oats and rye may be more beneficial to cardiovascular health than whole grain wheat [36]. While it is important to observe the effects of mixed-grain diets, as most people consume multiple types of grains, authors should report the specific types and relative amounts of individual grains.

There was also concern in the reporting of intervention compliance. Most studies used questionnaires or diaries but these are open to bias. One study using a biomarker found that in a whole grain intervention group, only approximately 40% of the subjects complied with the target amount of whole grain [37]. We identified 26 moderate or long duration trials that incorporated an independent biomarker of whole grain intake. These biomarkers included blood or urine concentrations of enterolactones [38,39,40,41,42,43] and/or alkylresorcinols [31,40,41,44,45,46,47,48,49,50,51,52]. Use of reliable biomarkers of intake strengthens a study’s design by removing potential reporting bias associated with self-reported compliance. It should be noted that there are some limitations to using biomarkers; alkylresorcinols, for example, are only found in wheat, rye, and, to a lesser extent, barley, while enterolactones are confounded by antibiotic intake and are increased with many plant foods [53]. In addition, most biomarkers have short half-lives, which means they generally only reflect recent intake [44], though cereal foods are usually eaten on a daily basis so this is less of a limiting factor. The issue of compliance is increasingly important, as we noted that more studies are moving away from completely controlled diet interentions, which can be expensive, and are instead relying on subjects to incorporate the intervention food(s) into their usual or normal diet.

The changing landscape of nutrition research makes whole grain research challenging to summarize and our evidence map highlights gaps in the research and study design considerations for future work. While there are many meta-analyses that focus on whole grains [4,54,55,56], there are fewer that focus on the health effects of specific grains other than oats [57,58,59,60]. However, we found that a large proportion of intervention studies administer whole wheat (21%) and a number of studies also focus on rye (15%), barley (8%) or brown rice (8%). This is similar to findings in a systematic review by Cho et al., stating that, “most RTCs and meta-analyses of RCTs did not capture the impact of major whole grains, such as wheat and corn, consumed in the United States” [61]. Therefore, more meta-analyses on these specific grains may be beneficial, especially in influencing policy and/or dietary recommendations [62]. Research on the health benefits of whole-grains should continue to grow with a particular focus on intervention studies examining less studied whole-grains such as millet, sorghum, quinoa, bulgur, amaranth, teff and triticale.

Evidence mapping is a dynamic method that allows for the ongoing addition of newly published literature. Thus, it allows us to capture the evolution of study design reporting practices in the whole-grain field. One limitation of this evidence map is that it does not include any data on the effect of whole grains on outcomes. However, systematically updating our whole grain database and corresponding evidence map will help to identify major gaps and/or areas with sufficient data on health benefits and, thus, provide the justification needed to move forward with a systematic review or meta-analysis. Detailed reporting of whole grain interventions is critical for facilitating summaries of the evidence, through systematic reviews, meta-analyses, or evidence mapping. Finally, researchers pursuing future work in the field must focus on standardizing whole grain reporting practices and study methodologies. Ensuring these best practices will allow for effective synthesis of study results in meta-analyses and, thereby, provide a stronger foundation to better inform nutrition scientists and public health policy.

## Figures and Tables

**Table 1:** Study design characteristics of whole grain intervention trials in humans, organized by trial duration.

Characteristic	Acute			Moderate (>1 Day––6 Weeks)			Long (>6 Weeks)		
	Total	RCT	Other ^1^	Total	RTC	Other ^2^	Total	RTC	Other ^3^
**Number of trials, *n***	118	101	17	63	57	6	32	31	1
Parallel, *n* (%)	-	1 (1)	-	-	20 (35)	-	-	26 (84)	-
Crossover, *n* (%)	-	100 (99)	-	-	37 (65)	-	-	1 (3)	-
**Duration, *n* (%)**									
≤ 1 day	118 (100)	101 (100)	17 (100)	-	-	-	-	-	-
< 1 week	-	-	-	10 (16)	7 (12)	3 (50)	-	-	-
1 to 6 weeks	-	-	-	53 (84)	50 (88)	3 (50)	-	-	-
6 to 12 weeks	-	-	-	-	-	-	25 (78)	24 (77)	1 (100)
>12 weeks	-	-	-	-	-	-	7 (22)	7 (23)	0 (0)
**Sample size, *n* (%)**									
≤10	39 (33)	29 (29)	10 (59)	4 (6)	2 (4)	2 (33)	0 (0)	0 (0)	0 (0)
11–25	58 (49)	54 (53)	4 (24)	27 (43)	23 (40)	4 (67)	4 (13)	4 (13)	0 (0)
26–50	16 (14)	15 (15)	1 (6)	18 (29)	18 (32)	0 (0)	9 (28)	8 (26)	1 (100)
>50	5 (4)	3 (3)	2 (12)	14 (22)	14 (25)	0 (0)	19 (59)	19 (61)	0 (0)
**Age group, *n* (%)**									
Adults (≥18 year)	115 (97)	98 (97)	17 (100)	60 (95)	54 (95)	6 (100)	31 (97)	31 (100)	0 (0)
Adolescents (12–17)	0 (0)	0 (0)	0 (0)	2 (3)	2 (4)	0 (0)	0 (0)	0 (0)	0 (0)
Children (3–11 year)	2 (2)	2 (2)	0 (0)	1 (2)	1 (2)	0 (0)	0 (0)	0 (0)	0 (0)
NR	1 (1)	1 (1)	0 (0)	0 (0)	0 (0)	0 (0)	1 (3)	0 (0)	1 (100)
**Sex, mean % male ± SD**	54 ± 27	55 ± 26	50 ± 32	44 ± 25	43 ± 23	49 ± 42	42 ± 21	40 ± 18	100 ± 0
**Baseline health, *n* (%)**									
Healthy	79 (67)	67 (66)	12 (71)	30 (48)	26 (46)	4 (67)	4 (13)	4 (13)	0 (0)
Overweight/Obese	19 (16)	18 (18)	1 (6)	19 (30)	18 (32)	1 (17)	19 (59)	18 (58)	1 (100)
T1DM	2 (2)	1 (1)	1 (6)	0 (0)	0 (0)	0 (0)	0 (0)	0 (0)	0 (0)
T2DM	18 (15)	15 (15)	3 (18)	5 (8)	3 (5)	2 (33)	4 (12)	4 (13)	0 (0)
MetS (≥1 criteria) ^4^	8 (7)	7 (7)	1 (6)	20 (32)	19 (33)	1 (17)	15 (47)	14 (45)	1 (100)
Digestive Issues	3 (3)	2 (2)	1 (6)	3 (5)	3 (5)	0 (0)	1 (3)	1 (3)	0 (0)
**Region, *n* (%)**									
Europe	61 (52)	54 (53)	7 (41)	28 (44)	24 (42)	4 (67)	17 (53)	17 (55)	0 (0)
North America	36 (31)	33 (33)	3 (18)	21 (33)	20 (35)	1 (17)	8 (25)	8 (26)	0 (0)
Asia	12 (10)	5 (5)	7 (41)	10 (16)	9 (16)	1 (17)	6 (19)	5 (16)	1 (100)
Australia	6 (5)	6 (6)	0 (0)	3 (5)	3 (5)	0 (0)	0 (0)	0 (0)	0 (0)
Africa	2 (2)	2 (2)	0 (0)	0 (0)	0 (0)	0 (0)	0 (0)	0 (0)	0 (0)
South America	0 (0)	0 (0)	0 (0)	1 (2)	1 (2)	0 (0)	0 (0)	0 (0)	0 (0)
Multiple	1 (1)	1 (1)	0 (0)	0 (0)	0 (0)	0 (0)	1 (3)	1 (3)	0 (0)
**Controls ^5^, *n* (%)**									
Refined grain	98 (74)	86 (75)	12 (67)	47 (71)	44 (75)	3 (43)	22 (69)	21 (68)	1 (100)
Usual diet	0 (0)	0 (0)	0 (0)	2 (3)	1 (2)	1 (14)	5 (16)	5 (16)	0 (0)
Matched, no WG ^6^	4 (3)	3 (3)	1 (6)	4 (6)	4 (7)	0 (0)	2 (6)	2 (6)	0 (0)
Other ^7^/unspecified	21 (16)	17 (15)	4 (22)	6 (9)	6 (10)	0 (0)	3 (9)	3 (10)	0 (0)
Crossover of WG only ^8^	10 (8)	9 (8)	1 (6)	4 (6)	4 (7)	0 (0)	0 (0)	0 (0)	0 (0)
No control arm	0 (0)	0 (0)	0 (0)	3 (5)	0 (0)	3 (43)	0 (0)	0 (0)	0 (0)

RCT = randomized controlled trial; NR = not reported; SD = standard deviation; MetS = metabolic syndrome; WG = whole grain; ^1–3^ Other study designs include: ^1^ crossover, unspecified randomization (*n* = 14); parallel, unspecified randomization (*n* = 2); single arm (*n* = 1); ^2^ single arm with no control (*n* = 3) and single arm, with control period (*n* = 3); ^3^ unspecified randomization, crossover (*n* = 1); ^4^ Have metabolic syndrome or at least one criteria of metabolic syndrome (hyperlipidemia, hypertension, or hyperglycemia); ^5^ Note that some trials included more than one control arm (total control arms for acute, moderate and long duration trials are *n* = 133, 66 and 32, respectively); ^6^ Identical intervention as treatment arm(s) except without whole grain ingredient(s) (e.g., “controlled diet alone,” “yogurt without oats”); ^7^ Other controls include bran, sucrose and unspecified controls (ex: “placebo”); ^8^ Crossover studies of different whole grain types, without a non-whole grain arm.

**Table 2:** Reporting practice in moderate (>1 day to 6 weeks) and long (>6 weeks) duration whole grain intervention trials in humans.

Reporting Criteria	*n* (%)
**Reporting of dose**	
Amount of grain	52 (55)
Amount of food/product	34 (36)
Not reported ^1^	9 (9)
**Reported a definition of whole grain**	
Referenced established definition ^2^	10 (12)
Described WG or WG food	12 (14)
Not reported	61 (73)
**Reported distribution of grain types** ^3^	18 (46)
**Control**	
Refined grain	69 (70)
Usual diet	7 (7)
Other ^4^	22 (22)
**Adherence** ^5^	
Questionnaires/Records	69 (73)
Food weighing or observation	14 (15)
Biomarkers	26 (22)
Not reported	19 (20)

^1^ Either no dose reported (*n* = 6) or the dose was not clearly reported as amount of grain versus amount of food (*n* = 3); ^2^ Among studies published after 1999 (*n* = 83), the year of the first published definition by AACCI; AACCI 1999 (*n* = 2), AACCI 2005 (*n* = 1), AACCI 2013 (*n* = 2); Dietary Guidelines for Americans (DGA) 2005 (*n* = 1), DGA 2010 (*n* = 1); HealthGrain Forum 2014 (*n* = 4); Proceedings of Nutrition Society 2006 (*n* = 1); note that two publications referenced more than one established definition; ^3^ Among interventions that provided a variety of WGs simultaneously *n* = 39; ^4^ Other controls included bran (*n* = 4), sucrose (*n* = 1), unspecified low-fiber cereal (*n* = 1), “placebo” (*n* = 1), standard dietary advice for Type 2 Diabetes (*n* = 2), matched intervention without addition of WG (*n* = 6), cross-over of whole grain only (*n* = 4), or no control arm (*n* = 3); ^5^ Note that some studies used more than one category of adherence measure and therefore are counted in more than one category.

## Data Availability

The database of whole grain intervention studies is not publicly available at this time; however, all data in the database are sourced from published articles.

## Supplementary Materials

The following are available online at http://www.mdpi.com/2072-6643/10/8/1052/s1, Table S1: Search terms for whole grain intervention studies, Table S2: Whole grain descriptions in moderate (>1 day to 6 weeks) and long (>6 weeks) duration intervention trials, Figure S1: Number of whole grain interventions published from 1977 to 2017, Figure S2: Number of whole grain interventions by reported outcomes 1977–2017, Figure S3: Number of whole grain interventions by grain types studied 1977–2017.
