# Supplementary material for: Evaluating Whole Grain Intervention Study Designs and Reporting Practices Using Evidence Mapping Methodology
Source: Nutrients. 2018 Aug 9;10(8):1052. doi: 10.3390/nu10081052 (PMC6115963; doi:10.3390/nu10081052)
Supplement: Supplementary file 1 [file nutrients-10-01052-s001.zip › Supplementary materials/Sawicki_Supplemental Materials Revised_Evaluating Whole Grain Interventions_Nutrients Submission.docx]

**Supplementary Materials: Evaluating Whole Grain Intervention Study Designs and Reporting Practices using Evidence Mapping Methodology**

Caleigh M. Sawicki^1^, Kara A. Livingston^1^, Alastair B. Ross^2^, Paul F. Jacques^1^, Katie Koecher^3^, Nicola M. McKeown^1^

**Table S1. Search terms for whole grain intervention studies**

| The following search terms for whole grains were used to identify publications for the database: |
| --- |
| whole grain* OR wholegrain* OR whole-grain* OR wholemeal OR whole meal OR whole-meal OR wholewheat OR whole wheat OR whole-wheat OR brown rice OR wild rice OR purple rice OR black rice OR red rice OR whole rice OR whole barley OR hulled barley OR hull-less barley OR whole corn OR popcorn OR whole rye OR whole oat* OR oat* OR millet* OR fonio OR sorghum OR milo OR teff OR triticale OR amaranth OR buckwheat OR quinoa OR kaniwa OR canihua OR spelt OR emmer OR faro OR farro OR einkorn OR kamut OR durum OR bulgur OR freekeh OR whole grain cereal* OR wholegrain cereal* |
| The following search terms were used to limit search results to intervention studies: |
| randomized controlled trial OR controlled clinical trial OR randomized OR placebo OR clinical trials as topic OR randomly OR trial OR Random Allocation OR Double-blind Method OR Single-Blind Method OR clinical trial OR placebos OR random$ comparative study OR Evaluation studies OR Cross-Over Studies OR latin square OR intervention studies OR dietary intervention |

**Table S2.** Whole grain descriptions in the moderate (>1 day to 6 weeks) and long (> 6 weeks) duration intervention trials

| **Year** | **Whole grain definitions or descriptions** | **Country** | **Cited** | **Ref (PMID)** |
| --- | --- | --- | --- | --- |
| 2002 | “including bran and germ as well as endosperm” | USA |  | 12144714 |
| 2002 | “natural form, with the bran and germ present” | USA |  | 11976158 |
| 2007 | “The whole-grain foods used were defined as containing a minimum of 50% whole grain per dry substance, including the starchy endosperm, germ, and bran, in mainly milled form.” | Sweden |  | 17513398 |
| 2008 | "A whole-grain product in this study is defined as a product containing all parts of the naked cereal kernel and >=50% of whole grain on a dry matter basis." | Sweden |  | 18400704 |
| 2008 | “WG cereals comprise three distinct physiological regions, the endosperm, germ, and bran” | UK |  | 17761020 |
| 2010 | “milled from all edible components of grains...refined flour, which consists mainly of the starchy endosperm” | Italy |  | 20438321 |
| 2010 | “The term ‘whole grain’ has been used to describe foods that contain more than 51 % whole grain in which the naturally occurring proportions of germ, bran and endosperm are retained” | UK | AACC 2005 | 20307353 |
| 2010 | “The whole grain cereals met the requirement that 51% of the ingredients were whole grain” | USA |  | 20302646 |
| 2011 | "The whole-grain foods used were defined as containing a minimum of 50% of whole grain per DM [dry matter], including the starchy endosperm, germ, and bran, in milled form" | Denmark |  | 22357746 |
| 2011 | "The WG component of foods was assumed to have all three major anatomical fractions of the cereal grain (bran, germ, and endosperm) in the same proportions found naturally." | Switzerland | AACC 2013* | 21272402 |
| 2011 | “the three principle components (bran, germ and endosperm) were present in amounts naturally found in the grain” | UK |  | 21554817 |
| 2012 | “bran, germ, and endosperm in the same proportions as the original cereal grain” | Canada |  | 21195592 |
| 2012 | “Whole grains are classified as the entire edible portion of seeds and kernels and wholegrain foods are defined as those containing 51% of wholegrain ingredients by weight per reference amount customarily consumed” | UK | Proceedings of the Nutrition Society 2006 | 22546716 |
| 2013 | "The wholegrain products used were defined as containing a minimum of 51% wholegrain per dry substance, including the starchy endosperm, germ, and bran, mainly in milled form" | Finland and Italy | HealthGrain 2014 | 23462537 |
| 2013 | "The WG component of foods was assumed to have all three major anatomical fractions of the cereal grain (bran, germ, and endosperm) in the same proportions found naturally as per the American Association of Cereal Chemists definition." | Switzerland | AACC 2013 | 23616503 |
| 2013 | “… whole grains as defined by the US Department of Health and Human Services and US Department of Agriculture…” | UK | DGA 2005 | 23668675 |
| 2014 | “WGs contain fibrous bran, starchy endosperm, and nutrient-rich germ. Removing the bran and germ, or “refining” the grain, reduces the fiber, vitamin, mineral, and phytochemical contents of the grain product”; “The American Association of Cereal Chemists and the Food and Drug Administration define WG as “the intact, ground, cracked, or flaked caryopsis, whose principal anatomical components—the starchy endosperm, germ and bran—are present in the same relative proportions as they exist in the intact caryopsis”; “WG products made from milled flour (eg, bread, pasta) were required to have >51% of dry weight from WG flour” | USA | DGA 2010 & AACC 1999 | 24944054 |
| 2015 | “Whole-grain intake can be particularly difficult to assess accurately, owing to lack of a uniform definition of whole-grain foods and to large variations in product content.” | Finland | HealthGrain 2014 | 26330144 |
| 2016 | “contain all parts of the grain and >50% WG wheat on a dry matter basis” | USA |  | 26043861 |
| 2017 | “Whole grain was defined based on the Health Grain Forum definition, that all parts of the grain were present in the products in their correct proportions, allowing for some minor losses of bran.”; “… each product had between 25% and 100% whole grain by weight.” | France | HealthGrain 2014 | 28085022 |
| 2017 | "the outer bran and germ portions of intact rice grains (i.e., brown rice) are removed to produce white rice, which primarily comprises starchy endosperm" | Japan |  | 28662074 |
| 2017 | “Whole grains are defined as the intact edible portion of the fruit of the cereal plant or the ground, cracked, flaked, or rolled fruit so long as the original proportions of the bran, endosperm, and germ are present in nearly the same proportions in the processed grain as were found in the intact grain.” | USA | HealthGrain 2014 & AACC 1999 | 28230784 |

*Not cited directly in this publication, but cited in another publication detailing the same trial

**Figure S1. Whole Grain Interventions Published from 1977 to 2017**

**Figure S2. Whole Grain Interventions by Reported Outcomes 1977-2017**

**Figure S3. Whole Grain Interventions by Grain Types Studied 1977-2017**
